# Supplementary material for: P38α MAPK-induced senescence in cranial suture progenitor cells promotes craniosynostosis
Source: Commun Biol. 2025 Dec 13;9:83. doi: 10.1038/s42003-025-09350-8 (PMC12820082; doi:10.1038/s42003-025-09350-8)
Supplement: Supplementary file 2 — Supplementary information [file 42003_2025_9350_MOESM2_ESM.pdf]

Supporting Information for

P38α MAPK-Induced Senescence in Cranial Suture Progenitor Cells Promotes Craniosynostosis

Zong Chen,[1,2,3,8] Zhiyou Chen,[4,8] Xinyan Chen,[1] Yingying Yue,[5] Yu Wang,[1] Xueying Hou,[6,7] Xiaoshuang Guo,[1] Chenzhi Lai,[1] Guodong Song,[1] and Xiaolei Jin[1,*]

[1]Department of Craniomaxillofacial Surgery, Plastic Surgery Hospital, Chinese Academy of Medical Sciences & Peking Union Medical College. Beijing, China

[2]Department of Plastic Surgery, Peking Union Medical College Hospital, Chinese Academy of Medical Sciences & Peking Union Medical College, Beijing, China

[3]Center for Regenerative Medicine & Plastic Surgery Research, Peking Union Medical College Hospital, Beijing, China

[4]Sichuan Provincial People's Hospital, University of Electronic Science and Technology of China, Chengdu, China

[5]Jishuitan Hospital, Capital Medical University, Beijing, China

[6]Department of Neurosurgery, Shengjing Hospital of China Medical University, Shenyang, China

[7]Institute of Health Sciences, China Medical University, Shenyang, China

[8]These authors contributed equally

*Correspondence: jinxiaolei@psh.pumc.edu.cn

Supplemental Information: **Figures S1–S8**
Table S1 KEY RESOURCES TABLE
Unedited blot images

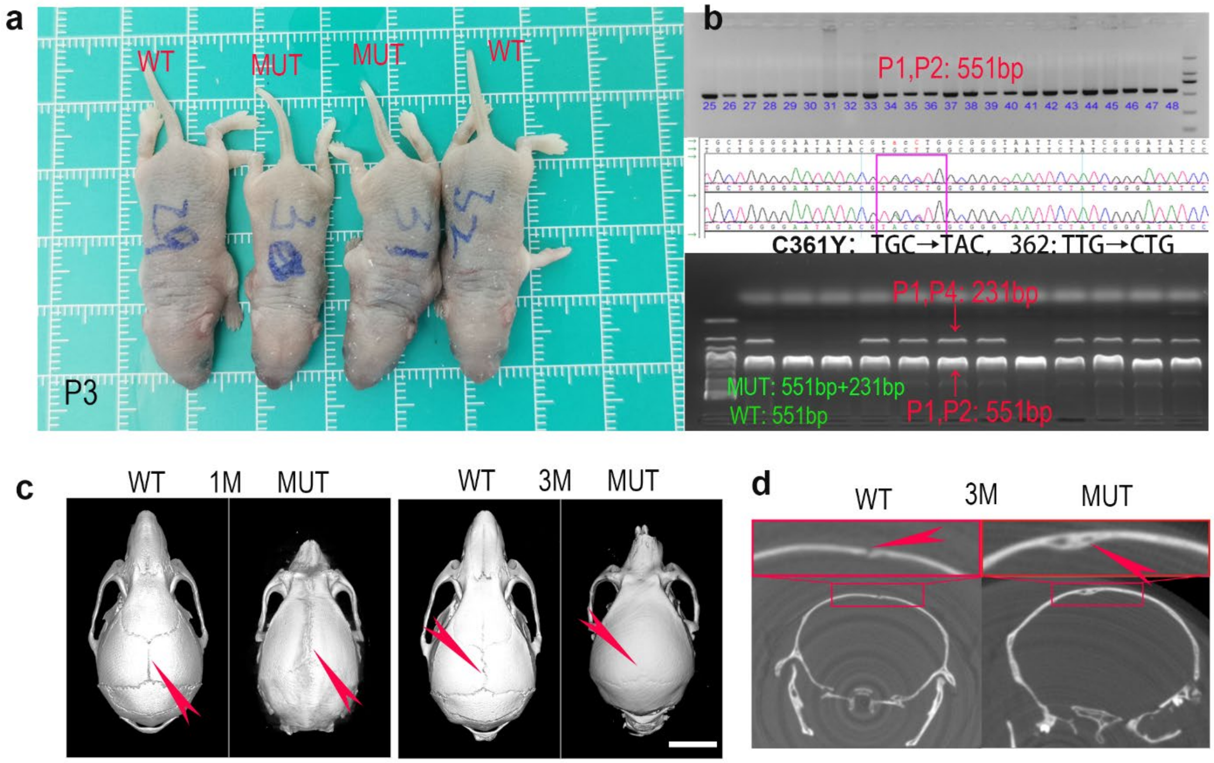

Figure S1. ***Fgfr2$^{C361Y/+}$*** **mice exhibit rapid coronal suture fusion and slower sagittal suture fusion**

(a) *Fgfr2$^{C361Y/+}$* mice were generated and identified using CRISPR-Cas9 technology and the mutant-type (MUT) mice were smaller in size compared to the wild-type (WT) mice.
(b) The images of mice genotyping that identified by DNA sequencing and agarose gel electrophoresis. The TGC→TAC mutation was designed at position 361 of the *Fgfr2*, and a TTG→CTG synonymous mutation was designed at position 362 to avoid secondary cleavage. With the sequence mutation, relevant primer sequences for genotyping were designed (See in the KEY RESOURCES TABLE).
(c) The µCT scanning images of skulls of MUT and WT mice. The sagittal sutures fused 3 months after birth. Scale bar: 5mm. N = 5 mice/group.
(d) The coronal X-ray images of MUT and WT mice at sagittal suture.

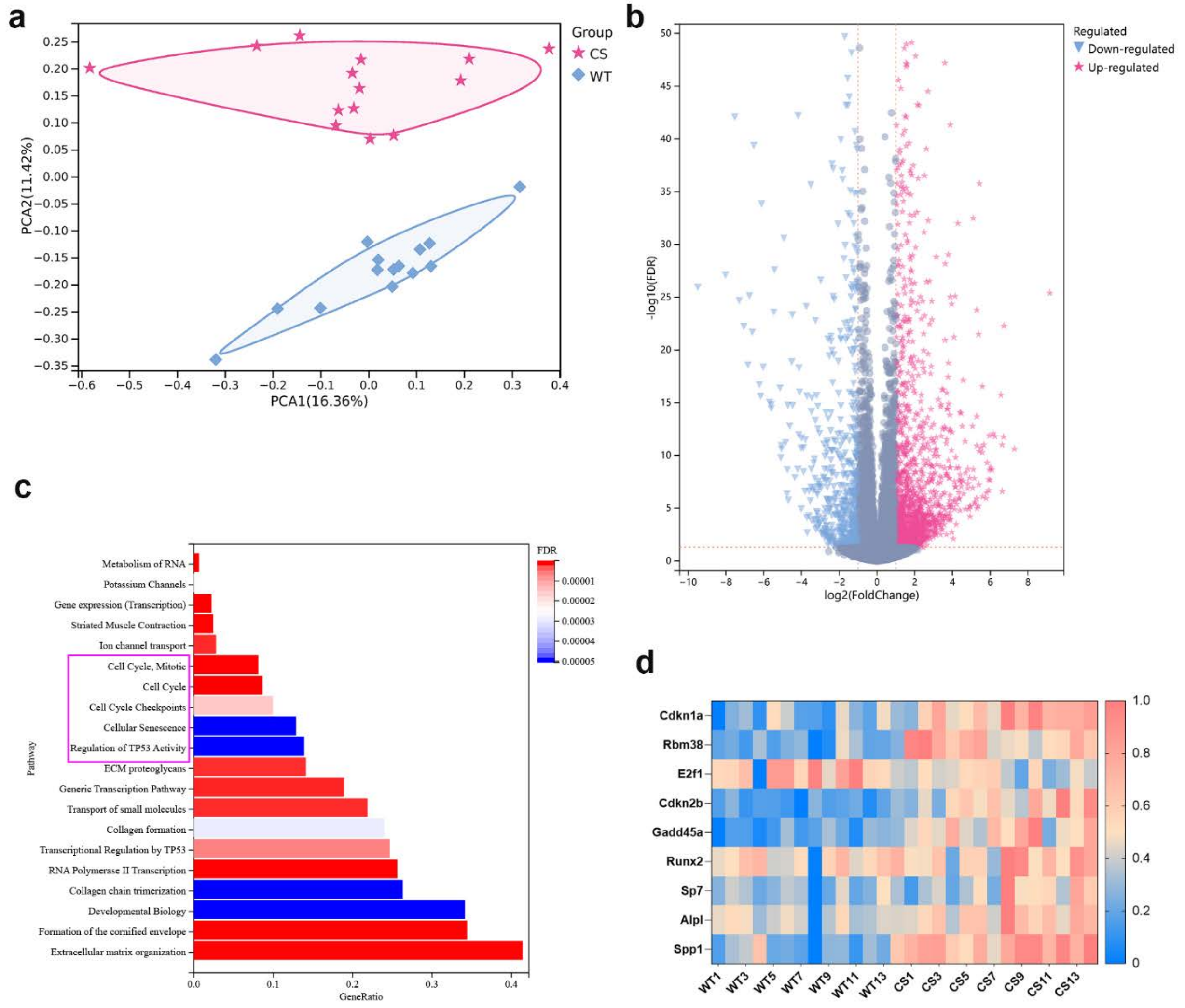

Figure S2. The bulk RNA sequencing indicates significant differences in transcriptional expression of coronal suture tissues between WUT and WT mice

(a) The plot of the principal component analysis (PCA) of bulk RNA sequencing. CS, Crouzon syndrome mice (MUT mice). N = 14 mice/group.

(b) The volcano plot of differential genes expression to bulk RNA sequencing.

(c) The enrichment analysis of top 20 Reactome pathways. FDR, false discovery rate.

(d) Heatmap of the expression levels of key genes in cell cycle pathway, p53 pathway and cell (osteogenic) differentiation pathway.

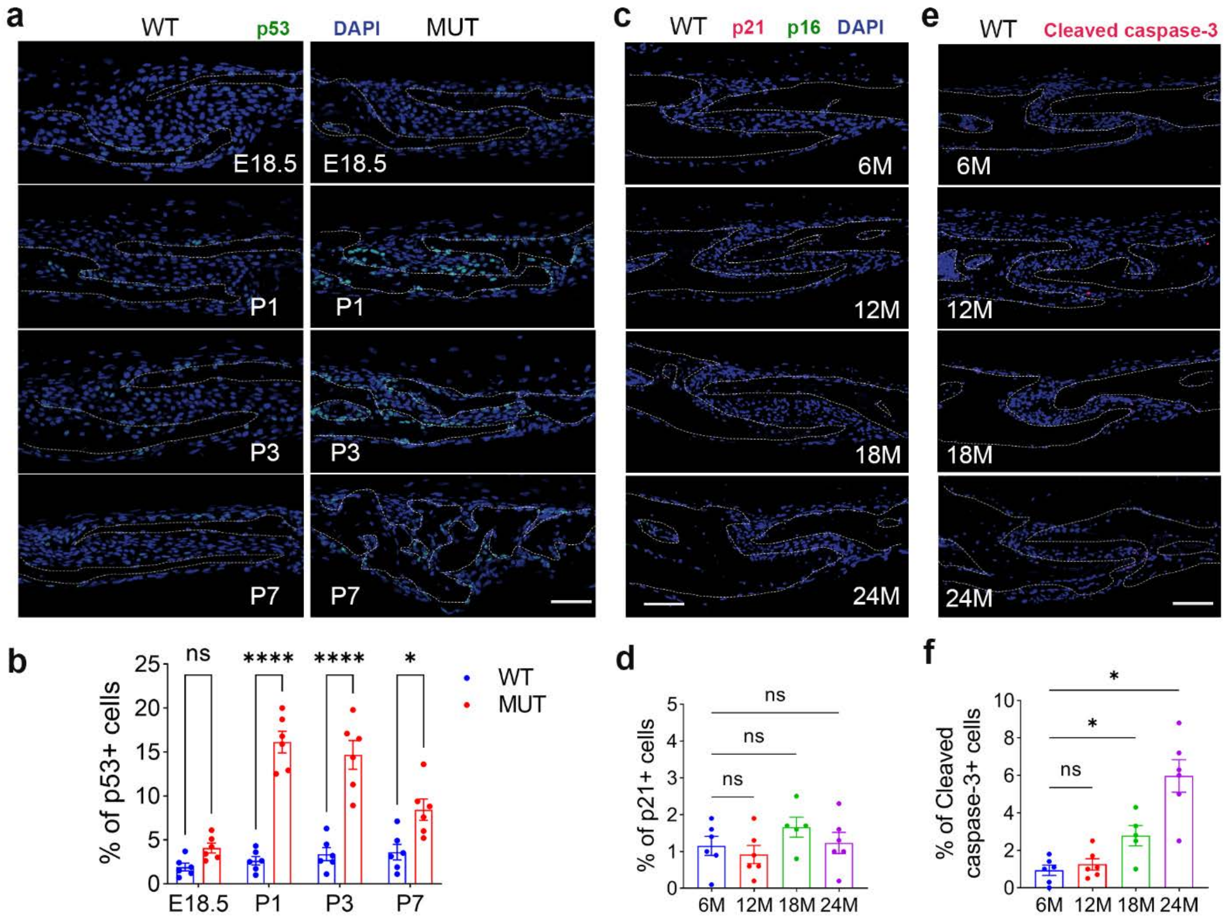

Figure S3. The decrease of coronal mesenchymal cells during WT mice aging is mainly caused by apoptosis

(a, b) The immunofluorescence staining images of p53 (a) and the quantification of p53 positive cells (b) in coronal sutures of MUT and WT mice. p53 was in green and DAPI was in blue. Scale bar: 100 μm. N = 6 sutures/group.

(c, d) The immunofluorescence staining images of p21 and p16 (c) and the quantification of p21 positive cells (d) in coronal sutures of 6M, 12M, 18M and 24M WT mice. p21 was in red, p16 was in green and DAPI was in blue. Scale bar: 100 μm. 6M, 12M, 24M, N = 6 sutures/group; 18M, N = 5 sutures/group.

(e, f) The immunofluorescence staining images of cleaved caspase-3 (e) and the quantification of cleaved caspase-3 positive cells (f) in coronal sutures of 6M, 12M, 18M and 24M WT mice. Cleaved caspase-3 was in green and DAPI was in blue. Scale bar: 100 μm. 6M, 12M, 24M, N = 6 sutures/group; 18M, N = 5 sutures/group.

All data are presented as mean ± SEM. For (b), two-way ANOVA was used, followed by Sidak's test. For (d) and (f), one-way ANOVA was used, followed by Dunnett's test. ***P* < 0.01, ****P* < 0.001, *****P* < 0.0001. ns, not significant.

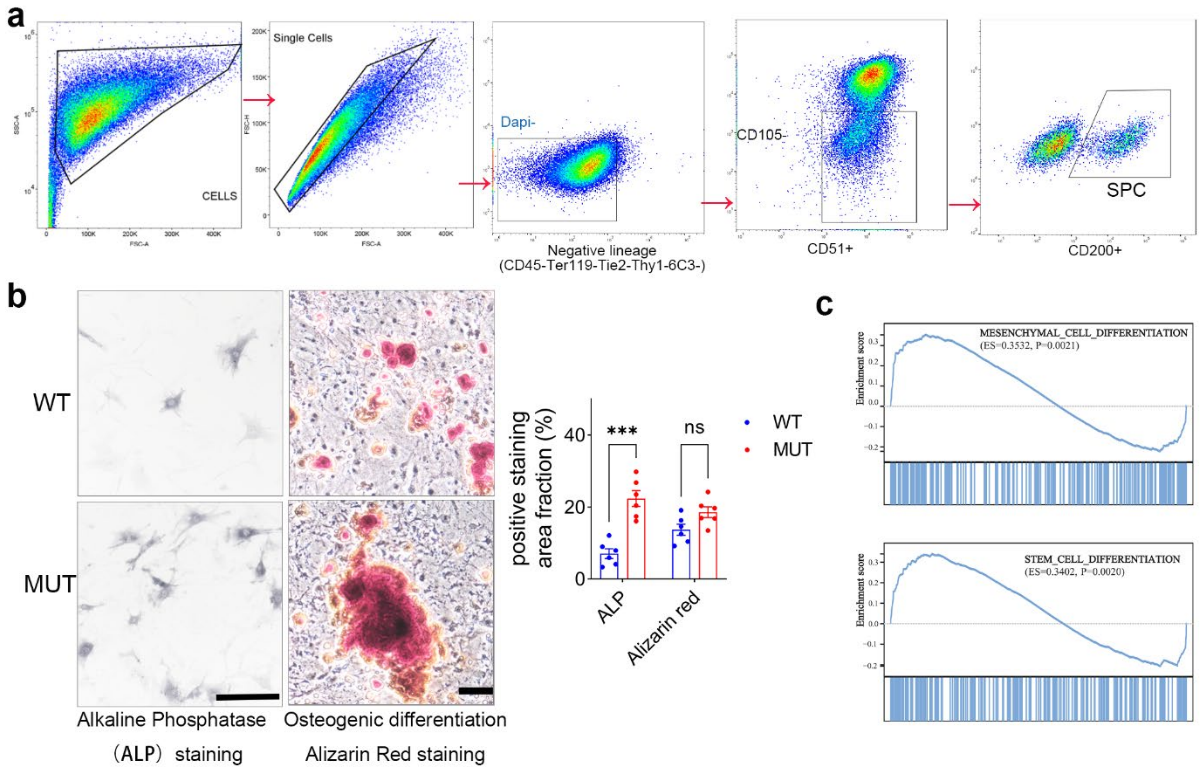

Figure S4. The CD51+CD200+CD105- Cells (suture progenitor cells, SPC) in coronal sutures of MUT and WT mice exhibit significant differences in osteogenic differentiation

(a) Gating strategy for identification and isolation of CD51+CD200+ SPC from coronal sutures. Total dissociated cells were fractionated by side scatter (SPC) and forward scatter (FSC) to discriminate single cells versus debris and doublets. Single cells were then fractionated by Dapi- and negative lineage (CD45-, Ter119-, Tie2-, Thy1- and 6C3-). The negative population was then further fractionated by CD51 and CD105. CD105-/CD51+ population was gated against CD200 for final isolation of SPC (CD51+CD200+CD105-CD45-Ter119-Tie2-Thy1-6C3-).

(b) The staining images and the quantification of positive staining area percentage of alkaline phosphatase (osteogenic induction for 3 to 5 days) and osteogenic differentiation (induction for 2 weeks) of WT and MUT SPC. Scale bar: 100 µm. N = 6 per group.

(c) The GSEAs specific to “Stem Cell Differentiation” and “Mesenchymal Differentiation” for bulk RNA sequencing data from coronal sutures of MUT mice and WT mice, N = 14 mice/group.

All data are presented as mean ± SEM. For (b), two-way ANOVA was used, followed by Sidak's test. $**P < 0.01$, $***P < 0.001$. ns, not significant.

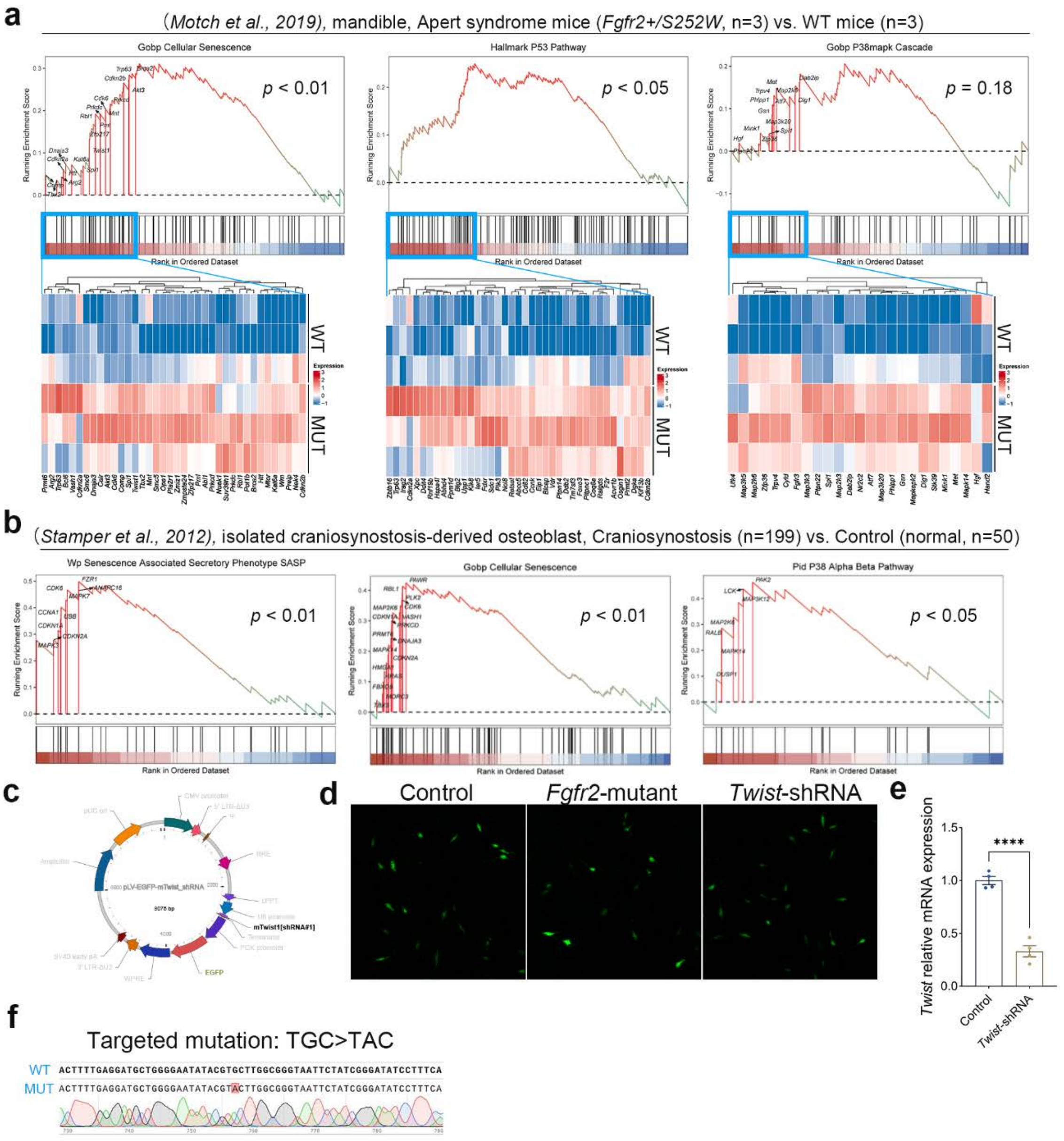

Figure S5. p38 and p53 activation, as well as cellular senescence, are commonly observed in the osteoblasts or bone tissues of human and mice with craniosynostosis.

(a) The GSEAs specific to “Cellular Senescence”, “p53 Pathway” and “P38 MAPK Cascade” gene sets and heatmaps of core-enriched genes for bulk RNA sequencing data from mandible tissues of Apert syndrome mice (*Fgfr2*$^{+/S252W}$) and WT mice, N = 3 per group.

(b) The GSEAs specific to “Senescence Associated Secretory Phenotype”, “Cellular Senescence” and “P38 Alpha Beta Pathway” gene sets and heatmaps of core-enriched genes for bulk RNA sequencing data from suture tissues-derived osteoblast of normal control and patients with isolated craniosynostosis. N = 199 in craniosynostosis group, and N 50 in normal control group.

(c) Schematic diagram of lentiviral plasmid of pLenti-*Twist*_shRNA-EGFP.

(d) Representative image of EGFP fluorescence expression in MC3T3-E1 cells transfected with lentivirus.
(e) Quantitation of *Twist* mRNA levels in MC3T3-E1 cells transfected with lentivirus (pLenti-*Twist*_shRNA-EGFP). N = 4.
(f) Sequencing alignment result of the *Fgfr2*-mutant lentiviral plasmid.
Data are presented as mean ± SEM. For (e), two-tailed *t* test was used. ****P < 0.0001.

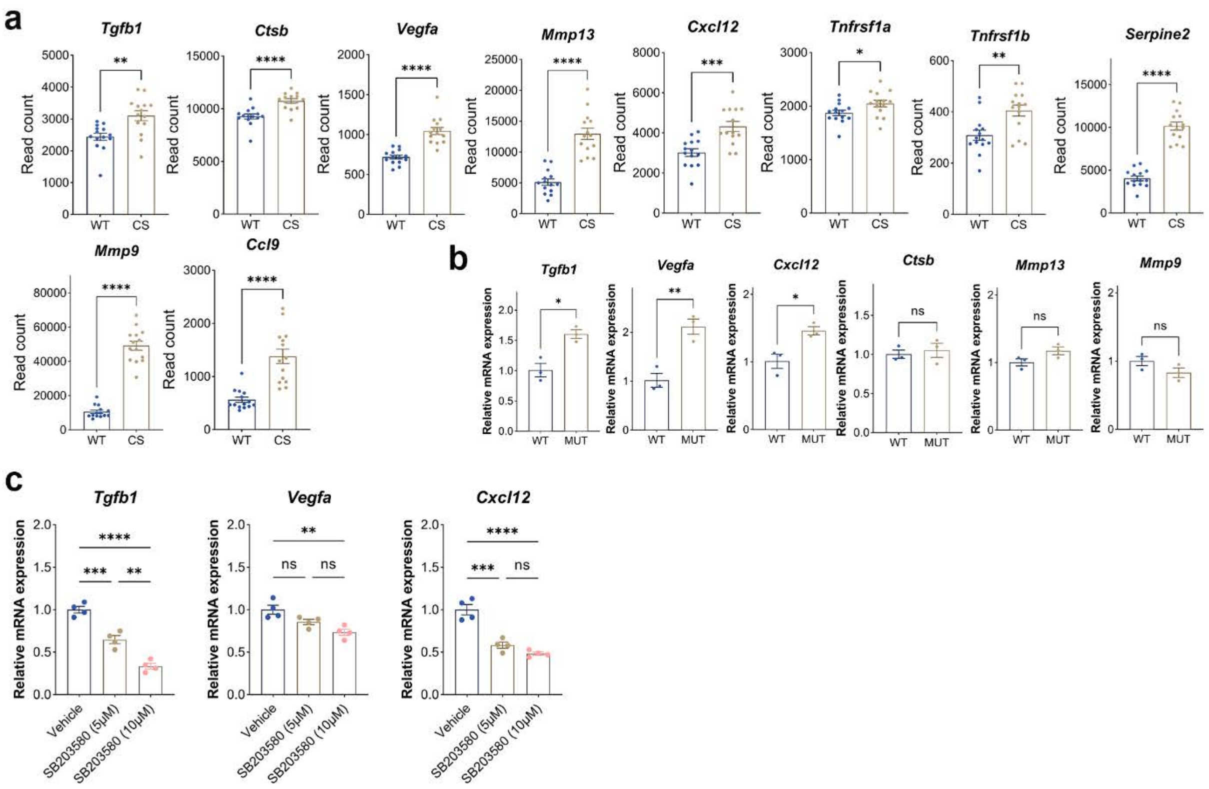

Figure S6. p38α MAPK mediates the senescence associated secretory phenotype (SASP) of MUT SPC

(a) Differential expression of SASPs based on bulk RNA sequencing. CS, Crouzon syndrome mice (MUT mice), N = 14 mice/group.

(b) Quantification of the key SASPs mRNA expression of WT and MUT SPC. N = 3.

(c) Quantification of the key SASPs mRNA expression of MUT SPC with inhibitors treatment. N = 4.

All data are presented as mean ± SEM. For (a) and (b), two-tailed *t* test was used. For (c), one-way ANOVA was used, followed by Tukey's test. **P* < 0.05, ***P* < 0.01, ****P* < 0.001, *****P* < 0.0001. ns, not significant.

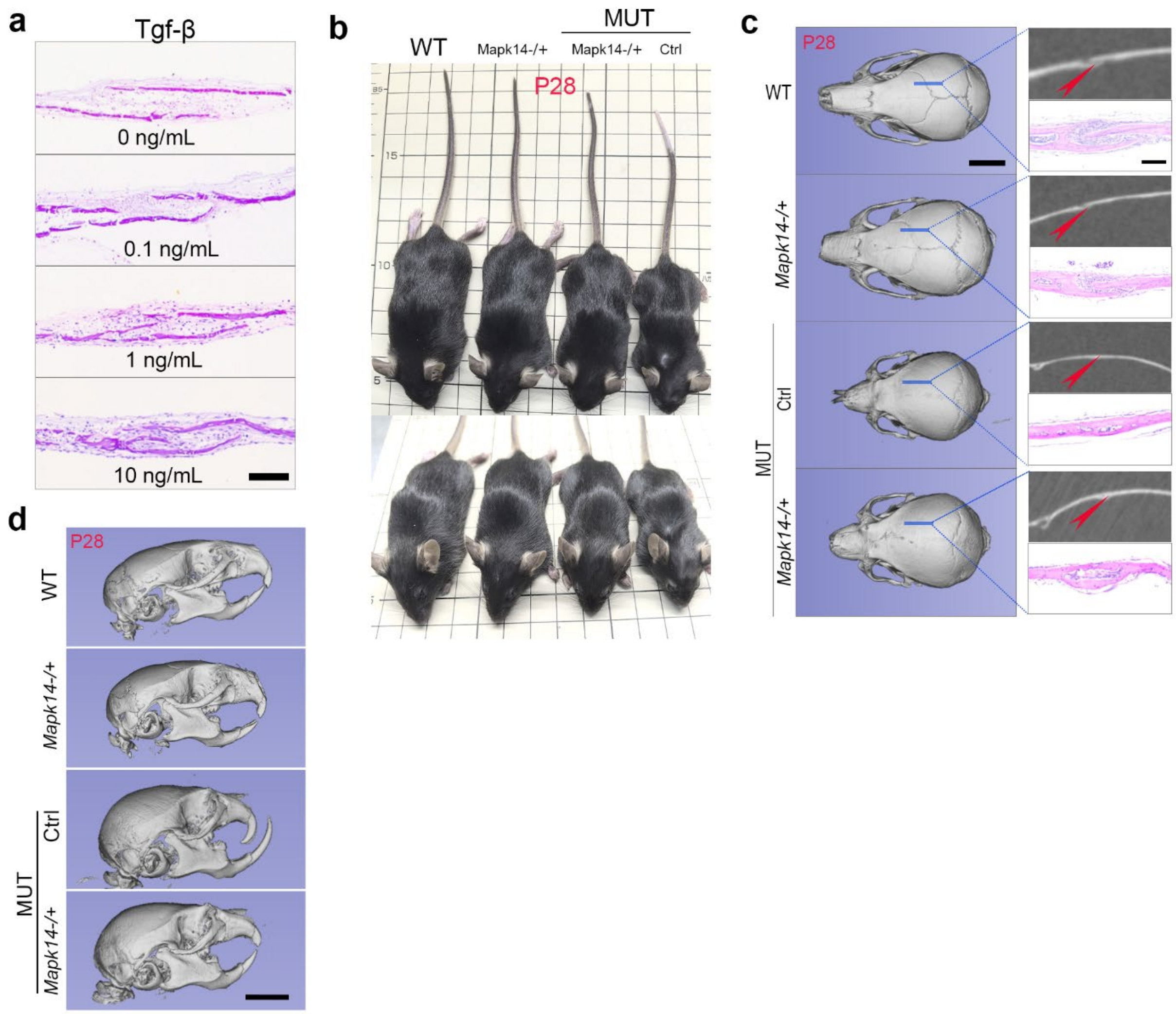

Figure S7. Genetic attenuation therapy of p38α MAPK mitigate the craniofacial deformities in MUT mice

(a) Representative H&E staining images of coronal sutures of the explants treated with Tgf-β1. Scale bar: 100 μm.

(b) The overall appearance images of WT and MUT mice with/without Mapk14-/+ knockout at P28.

(c) The μCT scanning images of skulls and H&E staining images of sutures from WT and MUT mice with/without Mapk14-/+ knockout. The red arrows indicated the fusion (or relief) of the coronal sutures in the sagittal plane of scanning images. Scale bar for μCT scanning images: 5 mm. Scale bar for H&E staining images: 100 μm. N = 4 to 7 mice/group.

(d) The μCT scanning images of side view of skulls of WT and MUT mice with/without Mapk14-/+ knockout at P28. Scale bar: 5 mm. N = 4 to 7 mice/group.

Data are presented as mean ± SEM. For (d), two-tailed *t* test was used. ****$P < 0.0001$.

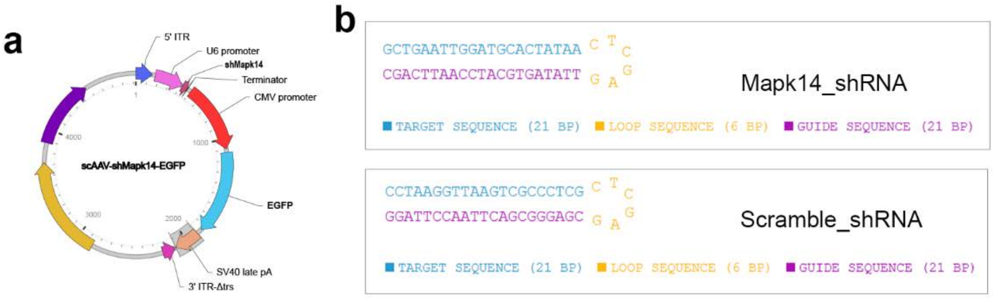

Figure S8. The scAAV-attenuation therapy of p38α MAPK mitigate the craniofacial deformities in MUT mice

(a) Schematic diagram of plasmid design for scAAV.

(b) The shRNA sequences and relevant information corresponding to Mapk14 and Scramble loaded with scAAV.

KEY RESOURCES TABLE (Table S1)

REAGENT OR RESOURCE	SOURCE	IDENTIFIER
**Antibodies**		
PE Anti-Mouse CD51	Elabscience	E-AB-F1235D
APC Anti-Mouse CD105	Elabscience	E-AB-F1233E
FITC Anti-Mouse CD200/OX2	Elabscience	E-AB-F1234C
Biotin Anti-Mouse CD90.2/Thy1.2	Elabscience	E-AB-F1094B
Biotin Anti-Mouse CD45	Elabscience	E-AB-F1122B
Biotin anti-mouse Ly-51/6C3	Biolegend	108303
Biotin anti-mouse CD202b /Tie-2	Biolegend	124005
Biotin Anti-Mouse TER-119	Elabscience	E-AB-F1125B
Purified Anti-Mouse CD16/32	Elabscience	E-AB-F0997A
Streptavidin PerCP-Cyanine5.5 Conjugate	eBioscience	45-4317-82
Rabbit Anti-p21	Abcam	ab188224, RRID: AB_2734729
Mouse Anti-p16	Santa Cruz Biotechnology	sc-1661, RRID: AB_628067
Mouse Anti-p53	Santa Cruz Biotechnology	sc-126, RRID: AB_628082
Rabbit Anti-Cleaved Caspase-3	Cell Signaling Technology	9661, RRID: AB_2341188
Rabbit Anti-Prrx1	Affinity Biosciences	DF4274, RRID: AB_2836625
Mouse Anti-Ctsk	Santa Cruz Biotechnology	sc-48353, RRID: AB_2087687
Mouse Anti-p21	Santa Cruz Biotechnology	sc-6246, RRID: AB_628073
Rabbit Anti-Phospho-p38	Cell Signaling Technology	4511, RRID: AB_2139682
Rabbit Anti-Phospho-Rb	Cell Signaling Technology	8516, RRID: AB_11178658
Rabbit Anti-Osteocalcin/Ocn	Bioss	bs-4917R, RRID: AB_2916189
Rabbit Anti-Tgf-β1	Abcam	ab215715, RRID: AB_2893156
Mouse Anti-Alp	Santa Cruz Biotechnology	sc-365765, RRID: AB_10842161
Rabbit Anti-Ki-67	Cell Signaling Technology	12202, RRID: AB_2620142
Rabbit Anti-p38	Cell Signaling Technology	9212, RRID: AB_330713
Rabbit Anti-Runx2	Cell Signaling Technology	12556, RRID: AB_2732805
Rabbit Anti-β-Actin	Cell Signaling Technology	4970, RRID: AB_2223172
Rabbit Anti-GAPDH	Cell Signaling Technology	2118, RRID: AB_561053
Goat Anti-Rabbit IgG, HRP Conjugate	Abcam	ab6721, RRID: AB_955447
Goat Anti-Mouse IgG, HRP Conjugate	Abcam	ab6789, RRID: AB_955439
Goat Anti-Rabbit IgG, Alexa Fluor 488	Abcam	ab150081, RRID: AB_2734747
Goat Anti-Rabbit IgG, Alexa Fluor 647	Abcam	ab150083, RRID: AB_2714032

Goat Anti-Mouse IgG, Alexa Fluor 488	Abcam	ab150117, RRID: AB_2688012
Goat Anti-Mouse IgG, Alexa Fluor 647	Abcam	ab150119, RRID: AB_2811129
**Bacterial and virus strains**		
scAAV-shRNA_Scramble-EGFP	OBiO Technology/this paper	N/A
scAAV-shRNA_Mapk14-EGFP	OBiO Technology/this paper	N/A
pLenti-shRNA_Twist-EGFP	OBiO Technology/this paper	N/A
pLenti-EGFP	OBiO Technology/*Yue et al.*	N/A
pLenti-Fgfr2_215_C361Y-EGFP	OBiO Technology/*Yue et al.*	N/A
**Chemicals, peptides, and recombinant proteins**		
Collagenase A	Roche	10103586001
HEPES	Gibco	15630-080
Penicillin-Streptomycin (10,000 U/mL)	Gibco	15140-122
Fetal Bovine Serum	VivaCell	C04001-500
PBS	Servicebio	G4202
DPBS	Gibco	14190144
DMEM	Corning	10-014-CV
Accutase	Gibco	A1110501
SB203580	MedChemExpress	HY-10256A
Pifithrin-α	MedChemExpress	HY-15484
SB525334	MedChemExpress	HY-12043
Tgf-β1 protein	MedChemExpress	HY-P7117
0.5M EDTA	Beyotime	ST069
DAPI	Thermo Scientific	62248
RNase A	Thermo Scientific	EN0531
7-AAD staining solution	BD	559925
OTC	Sakura Finetek	4583
EDTA decalcified solution	Biosharp	BL616B
Triton X-100	Beyotime	ST1723
Antibody dilution buffer	Beyotime	P0269
Mounting Medium with DAPI	Abcam	ab104139
Goat Serum	Beyotime	C0265
Paraformaldehyde fix solution	Beyotime	P0099
RIPA Lysis Buffer	Beyotime	P0013C
PVDF membrane	Millipore	ISEQ00010
ECL solution	Beyotime	P0018M

L-Ascorbic acid	MedChemExpress	HY-B0166
DMSO	MedChemExpress	HY-Y0320
TRIzol	Invitrogen	15596018CN
**Critical commercial assays**		
siRNA Reagent System	Santa Cruz Biotechnology	sc-45064
Senescence β-Galactosidase Staining Kit	Cell Signaling Technology	9860
Comet Assay Kit	Abbkine	KTA3040
Osteogenic Differentiation Kit	OriCell	MUXMX-90021
Adipogenic Differentiation Kit	OriCell	MUXMX-90031
Chondrogenic Differentiation Kit	OriCell	MUXMX-90031
Alkaline Phosphatase Color Development Kit	Beyotime	C3250S
E-Click EdU Cell Proliferation Assay Kit	Elabscience	E-CK-A378
Luciferase Reporter Gene Assay Kit	Beyotime	RG088S
Mouse VEGF ELISA Kit	Beyotime	PV957
Mouse CXCL12 ELISA Kit	Beyotime	PC201
Mouse TGF-β1 ELISA Kit	Beyotime	PT878
BCA protein assay kit	Beyotime	P0010
Two step immunohistochemical staining kit	Zsbio	PV-9000
HiScript III 1st Strand cDNA Synthesis Kit	Vazyme	R312-02
Taq Pro Universal SYBR Master Mix	Vazyme	Q712-02
**Deposited data**		
Bulk RNA sequencing	This paper	GSA: CRA018981
**Experimental models: Cell lines**		
SPC	This paper	N/A
MC3T3-E1	Pricella	C3078
**Experimental models: Organisms/strains**		
Mouse: C57BL/6J	Charles River Laboratories	219
Mouse: *Fgfr2*$^{C361Y/+}$	GemPharmatech/*Yue et al.*	N/A
Mouse: *Mapk14-/+*	Shanghai Model Organisms	NM-KO-225013
Mouse: *Mapk14-Flox*	Shanghai Model Organisms	NM-KO-2102209
Mouse: *Prrx1-Cre*	The Jackson Laboratory	005584
Mouse: *Fgfr2*$^{C361Y/+}$*; Mapk14-/+*	This paper	N/A

Mouse: *Fgfr2*$^{C361Y/+}$*; Prrx1*$^{cre/+}$*; Mapk14*$^{f/+}$	This paper	N/A
Mouse: *Fgfr2*$^{C361Y/+}$*; Prrx1*$^{cre/+}$*; Mapk14*$^{f/f}$	This paper	N/A
**Oligonucleotides**		
For genotyping of *Fgfr2*$^{C361Y/+}$, outer primer-forward: TGCAGTTGGAATCTCCTGATGG	Tsingke Biotechnology	N/A
For genotyping of *Fgfr2*$^{C361Y/+}$, outer primer-reverse: GCCCACTGAGTCAACAATTCAGC	Tsingke Biotechnology	N/A
For genotyping of *Fgfr2*$^{C361Y/+}$, inner primer-reverse: CGATAGAATTACCCGCCAGGT	Tsingke Biotechnology	N/A
For genotyping of *Mapk14+/-*, outer primer-forward: TGGGTTCAGTGCCTTCTTCC	Tsingke Biotechnology	N/A
For genotyping of *Mapk14+/-*, outer primer-reverse: TGTGTGTCAGCATGCAAAGC	Tsingke Biotechnology	N/A
For genotyping of *Mapk14+/-*, outer primer-reverse: CAGTGTCCTGATTTTCTACCCAGA	Tsingke Biotechnology	N/A
Primer: *Actb*-forward: CCTGTATGCCTCTGGTCGTA	Tsingke Biotechnology	N/A
Primer: *Actb*-reverse: CGCTCGTTGCCAATAGTGAT	Tsingke Biotechnology	N/A
Primer: *Tgfb*-forward: GCAACAACGCCATCTATG	Tsingke Biotechnology	N/A
Primer: *Tgfb*-reverse: CAAGGTAACGCCAGGAAT	Tsingke Biotechnology	N/A
Primer: *Vegfa*-forward: GGCTGCTGTAACGATGAA	Tsingke Biotechnology	N/A
Primer: *Vegfa*-reverse: CTGCTGTGCTGTAGGAAG	Tsingke Biotechnology	N/A
Primer: *Cxcl12*-forward: GTTCTTCGAGAGCCACAT	Tsingke Biotechnology	N/A
Primer: *Cxcl12*-reverse: CACTTGTCTGTTGTTGTTCT	Tsingke Biotechnology	N/A
Primer: *Mmp13*-forward: TGACCTCCACAGTTGACA	Tsingke Biotechnology	N/A
Primer: *Mmp13*-reverse: GTGCCAGAAGACCAGAAG	Tsingke Biotechnology	N/A

Primer: *Ctsb*-forward: GGACGCAACTTCTACAATG	Tsingke Biotechnology	N/A
Primer: *Ctsb*-reverse: GTATGGTAAGCAGCCTACA	Tsingke Biotechnology	N/A
Primer: *Bcl2*-forward: TCGTGACTTCGCAGAGAT	Tsingke Biotechnology	N/A
Primer: *Bcl2*-reverse: CAGAGACAGCCAGGAGAA	Tsingke Biotechnology	N/A
Primer: *Bax*-forward: CTGATGGCAACTTCAACTG	Tsingke Biotechnology	N/A
Primer: *Bax*-reverse: ATCTTCTTCCAGATGGTGAG	Tsingke Biotechnology	N/A
Control siRNA	Santa Cruz Biotechnology	sc-37007
Mapk14 siRNA	Santa Cruz Biotechnology	sc-29434
Mapk11 siRNA	Santa Cruz Biotechnology	sc-39117
MKK3 siRNA	Santa Cruz Biotechnology	sc-35908
MKK6 siRNA	Santa Cruz Biotechnology	sc-35914
**Recombinant DNA**		
pp53-TA-luc	Beyotime	D2223
pRL-TK	Beyotime	D2760
pscAAV-shRNA_Scramble-EGFP	VectorBuilder & OBiO Technology	VB010000
pscAAV-shRNA_Mapk14-EGFP	VectorBuilder & OBiO Technology	VB240207-1114
pscAAV-shRNA_Twist-EGFP	VectorBuilder & OBiO Technology	VB240207-1412
**Software and algorithms**		
3D slicer v5.6.2	https://download.slicer.org/	N/A
FlowJo v10.8.1	https://www.flowjo.com/solutions/flowjo/downloads	N/A
OpenComet v1.3	https://cometbio.org/download.html	N/A
LAS X v3.7.5	https://www.leica-microsystems.com/products/microscope-software/p/leica-las-x-ls/downloads/	N/A
ImageJ v1.54	https://cn.leica-microsystems.com.cn/service/software-download/las-x-lsr	N/A
R software v4.2.1	https://www.r-project.org/	N/A

Prism v9.5	https://www.graphpad-prism.cn/	N/A
**Other**		
40 µm Cell Strainer	Falcon	352340

Unedited blot images

Figure 1d

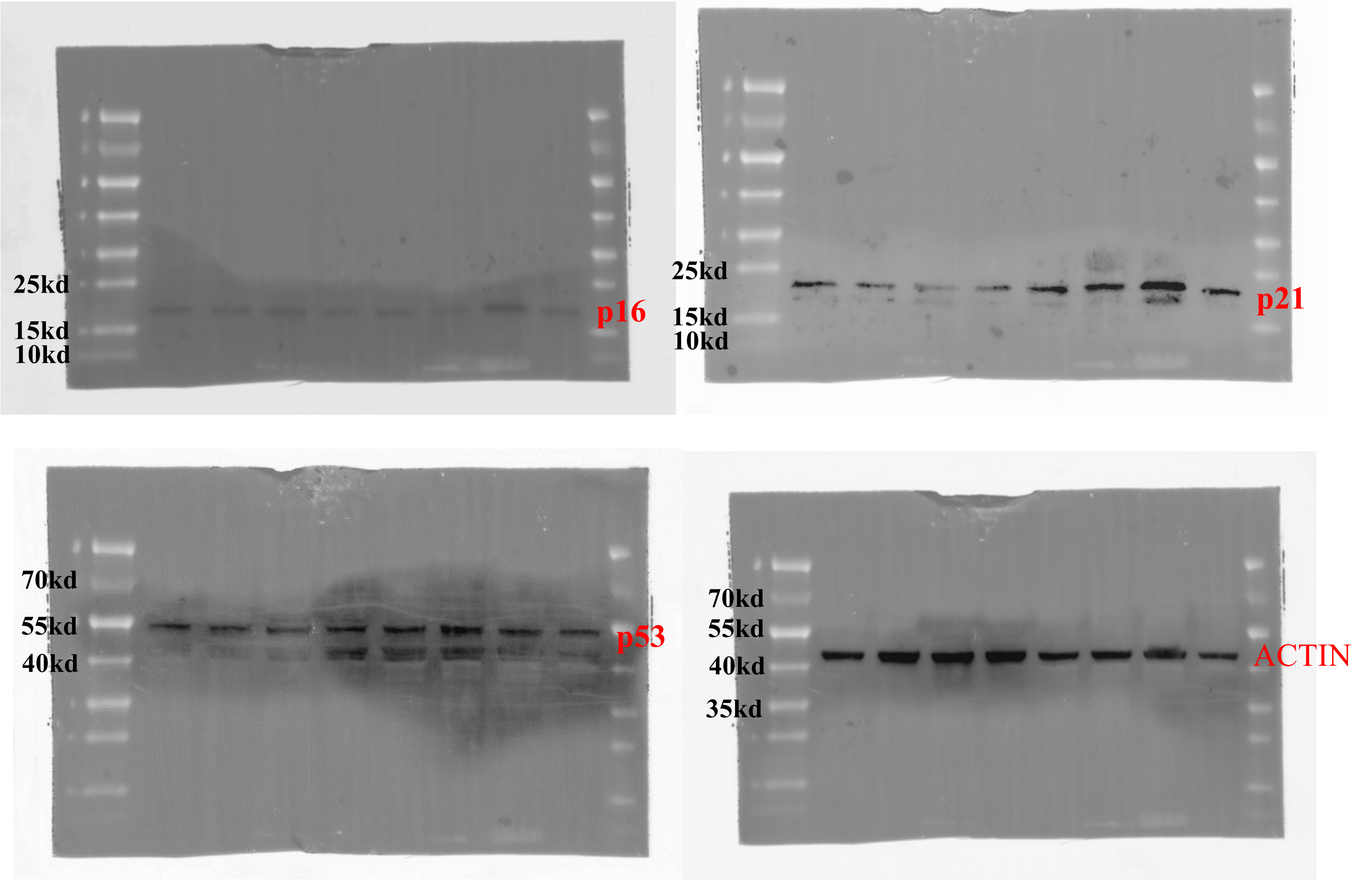

Figure 4b

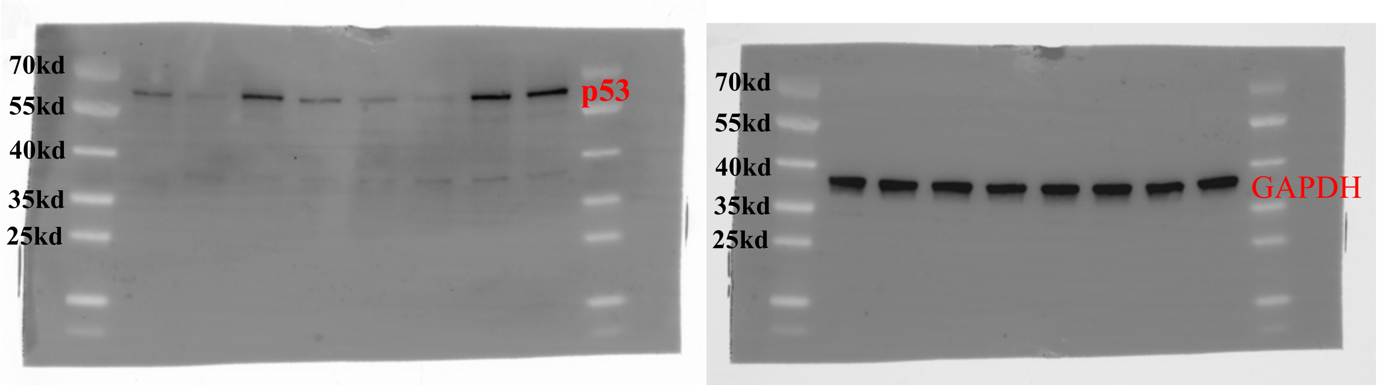

Thermo Scientific
Protein Ladder Cat.26619

Figure 4c

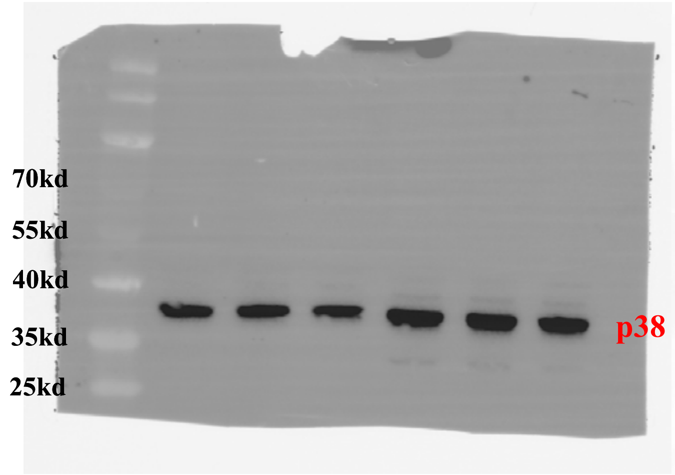

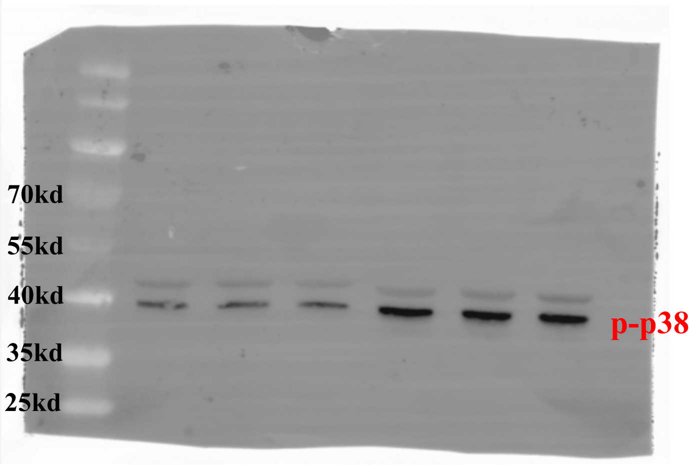

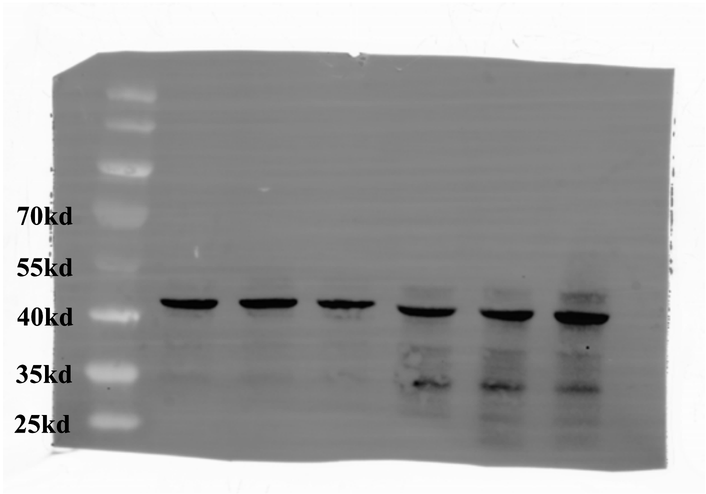

Thermo Scientific
Protein Ladder Cat.26619

Figure 4d

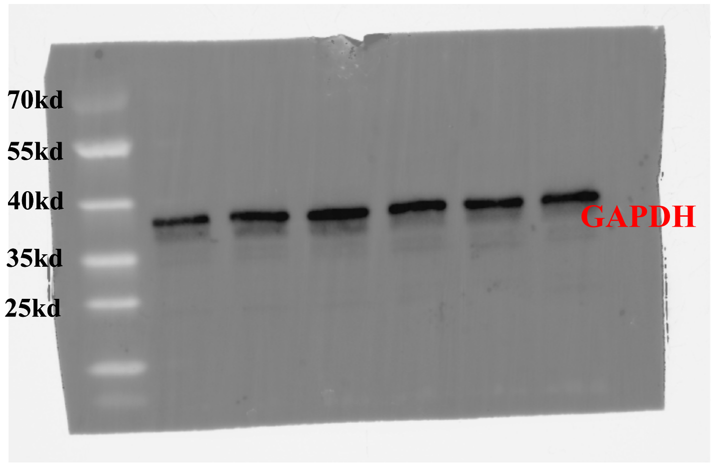

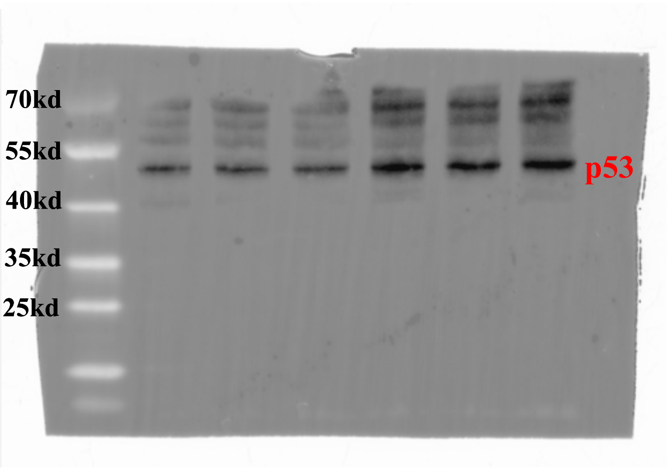

Thermo Scientific
Protein Ladder Cat.26619

Figure 4h

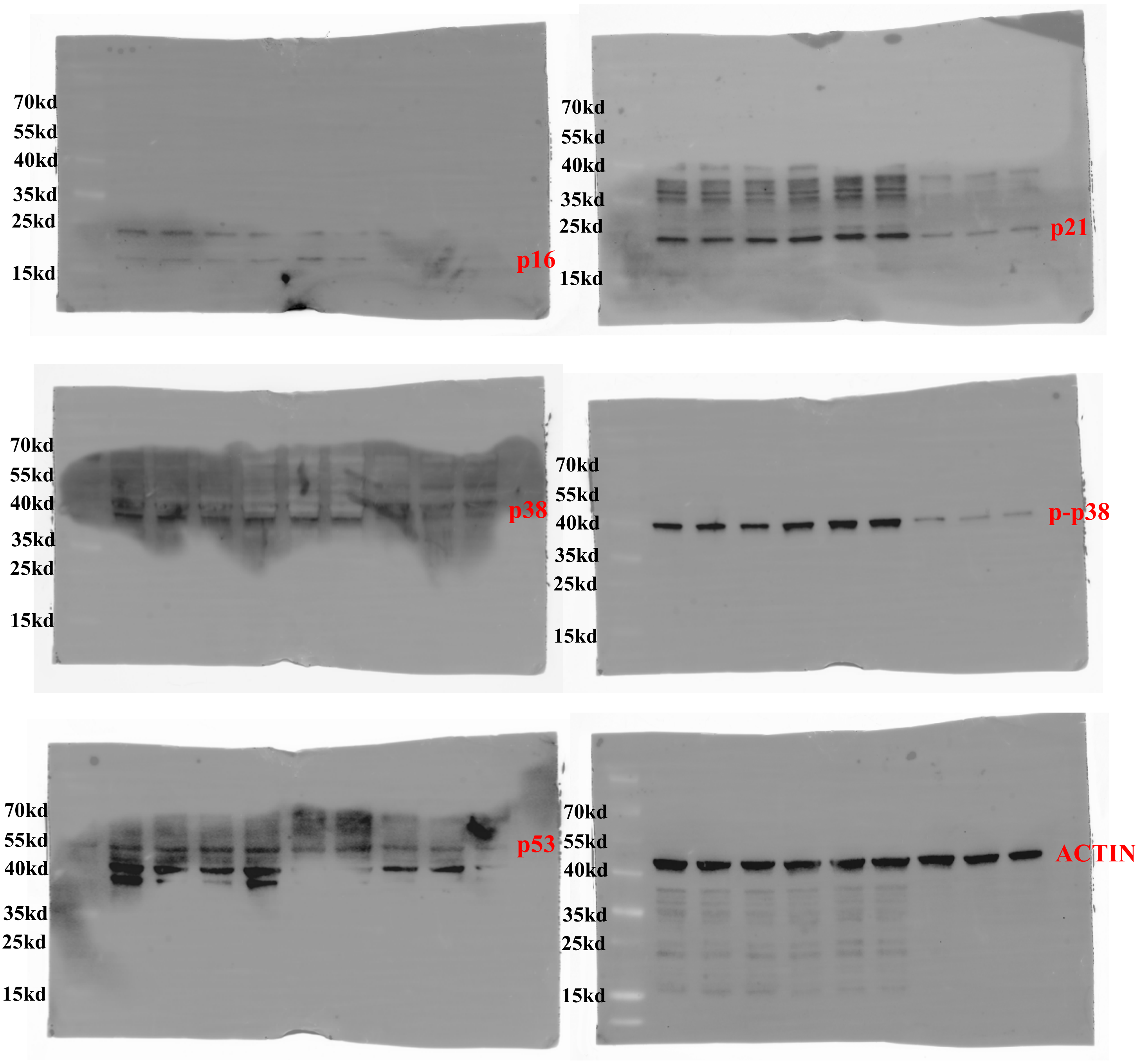

Thermo Scientific
Protein Ladder Cat.26619

Figure 5e

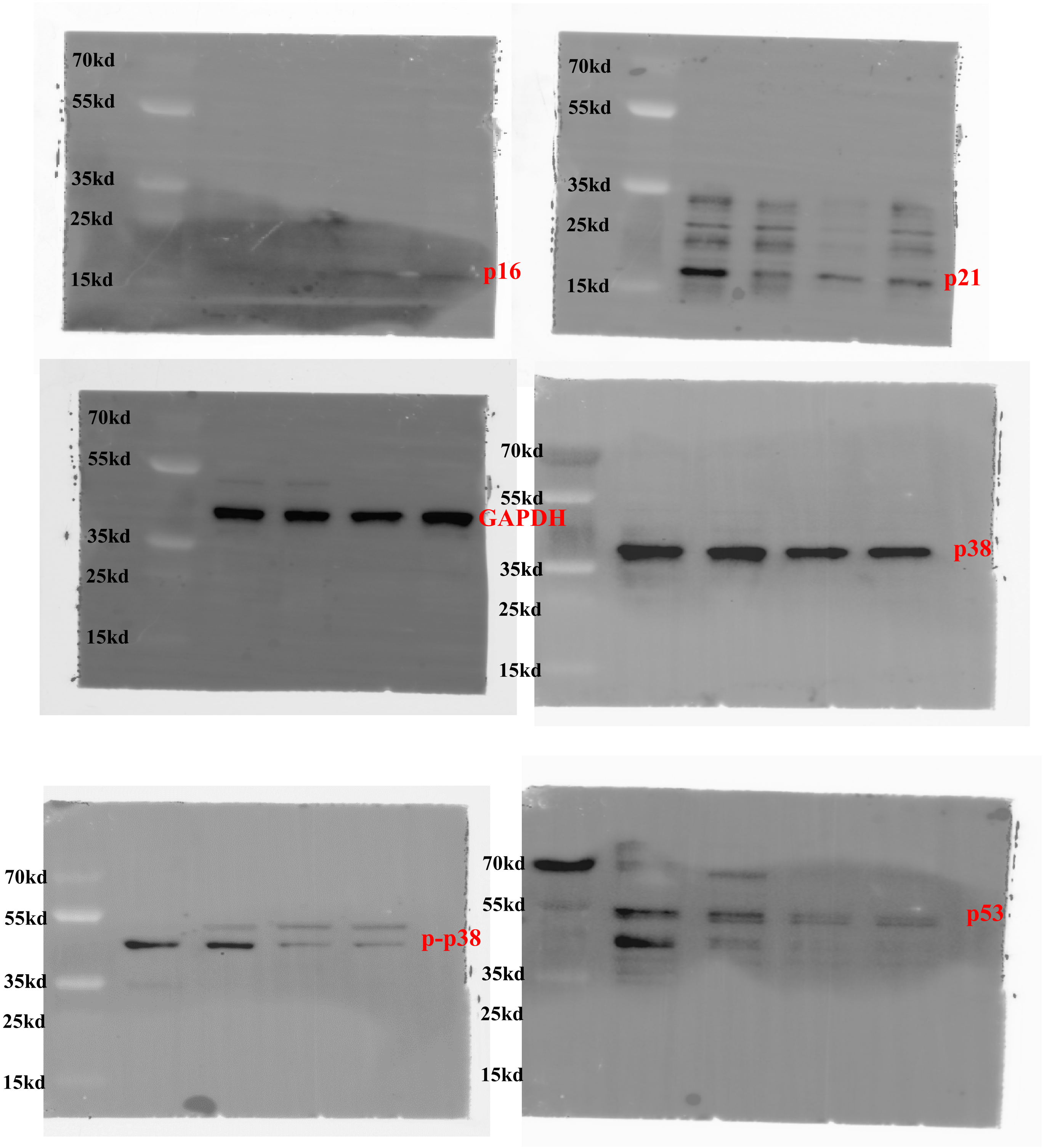

Thermo Scientific
Protein Ladder Cat.26616

Figure 6f

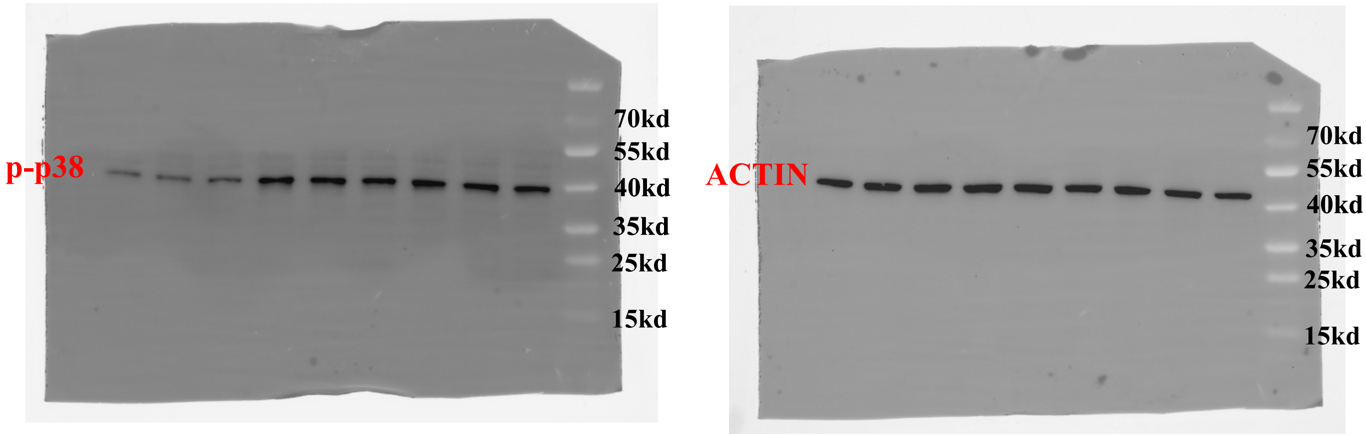

Thermo Scientific
Protein Ladder Cat.26619

Figure 8k

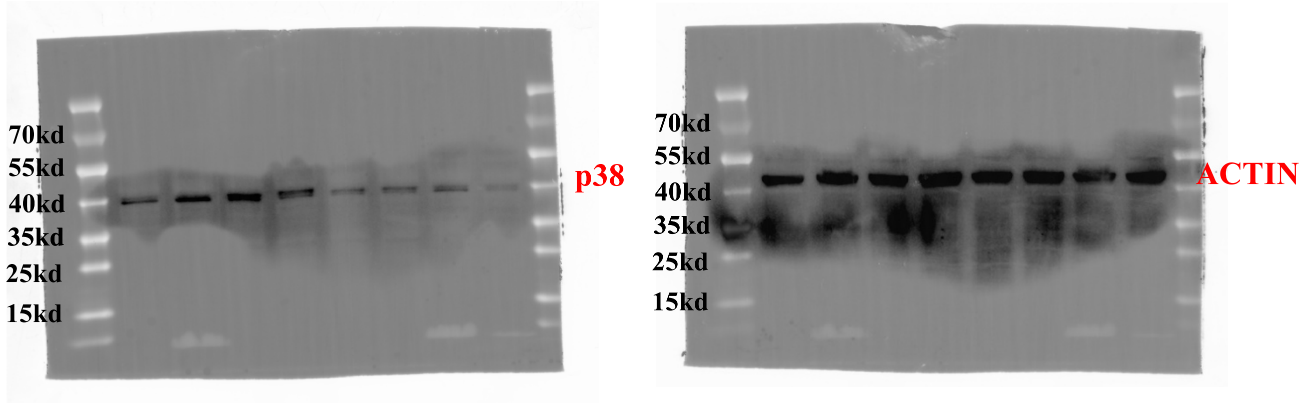

Thermo Scientific
Protein Ladder Cat.26619
