# Supplementary material for: P38α MAPK-induced senescence in cranial suture progenitor cells promotes craniosynostosis
Source: Commun Biol. 2025 Dec 13;9:83. doi: 10.1038/s42003-025-09350-8 (PMC12820082; doi:10.1038/s42003-025-09350-8)
Supplement: Supplementary file 5 — Reporting summary [file 42003_2025_9350_MOESM5_ESM.pdf]

nature portfolio

Corresponding author(s): Xiaolei Jin

Last updated by author(s): Aug 15, 2025

Reporting Summary

Nature Portfolio wishes to improve the reproducibility of the work that we publish. This form provides structure for consistency and transparency in reporting. For further information on Nature Portfolio policies, see our Editorial Policies and the Editorial Policy Checklist.

Statistics

For all statistical analyses, confirm that the following items are present in the figure legend, table legend, main text, or Methods section.

n/a	Confirmed	
☐	☒	The exact sample size (n) for each experimental group/condition, given as a discrete number and unit of measurement
☐	☒	A statement on whether measurements were taken from distinct samples or whether the same sample was measured repeatedly
☐	☒	The statistical test(s) used AND whether they are one- or two-sided<br>*Only common tests should be described solely by name; describe more complex techniques in the Methods section.*
☒	☐	A description of all covariates tested
☐	☒	A description of any assumptions or corrections, such as tests of normality and adjustment for multiple comparisons
☐	☒	A full description of the statistical parameters including central tendency (e.g. means) or other basic estimates (e.g. regression coefficient) AND variation (e.g. standard deviation) or associated estimates of uncertainty (e.g. confidence intervals)
☐	☒	For null hypothesis testing, the test statistic (e.g. F, t, r) with confidence intervals, effect sizes, degrees of freedom and P value noted<br>*Give P values as exact values whenever suitable.*
☒	☐	For Bayesian analysis, information on the choice of priors and Markov chain Monte Carlo settings
☒	☐	For hierarchical and complex designs, identification of the appropriate level for tests and full reporting of outcomes
☒	☐	Estimates of effect sizes (e.g. Cohen's d, Pearson's r), indicating how they were calculated

Our web collection on statistics for biologists contains articles on many of the points above.

Software and code

Policy information about availability of computer code

Data collection	Reconstruction and analysis of Cranial imaging were conducted using the 3D Slicer software (v5.6.2) and the PerkinElmer analysis system. The ImageJ software (Fiji package; v1.54) was used to analyze the protein bands. Fluorescent imaging was performed using a Leica SP8 confocal imaging system, and data processing was performed using the LAS X software (v3.7.5).
Data analysis	The FlowJo software (v10.8.1) was used for data analyses from flow cytometry. Comet assay data processing was performed using the OpenComet software (v1.3). Differential expression analysis of RNA-seq data was performed using the DESeq2 R package, and enrichment analysis and plotting were performed using the clusterProfiler package. Statistical analysis was performed using GraphPad Prism (v9.5.0).

For manuscripts utilizing custom algorithms or software that are central to the research but not yet described in published literature, software must be made available to editors and reviewers. We strongly encourage code deposition in a community repository (e.g. GitHub). See the Nature Portfolio guidelines for submitting code & software for further information.

Data

Policy information about availability of data

All manuscripts must include a data availability statement. This statement should provide the following information, where applicable:

- Accession codes, unique identifiers, or web links for publicly available datasets
- A description of any restrictions on data availability
- For clinical datasets or third party data, please ensure that the statement adheres to our policy

• Bulk RNA-seq data have been deposited at GSA (Genome Sequence Archive in National Genomics Data Center, https://ngdc.cncb.ac.cn/gsa/) at GSA: CRA018981 and are publicly available as of the date of publication.

Research involving human participants, their data, or biological material

Policy information about studies with human participants or human data. See also policy information about sex, gender (identity/presentation), and sexual orientation and race, ethnicity and racism.

Reporting on sex and gender	n/a
Reporting on race, ethnicity, or other socially relevant groupings	n/a
Population characteristics	n/a
Recruitment	n/a
Ethics oversight	n/a

Note that full information on the approval of the study protocol must also be provided in the manuscript.

Field-specific reporting

Please select the one below that is the best fit for your research. If you are not sure, read the appropriate sections before making your selection.

☒ Life sciences ☐ Behavioural & social sciences ☐ Ecological, evolutionary & environmental sciences

For a reference copy of the document with all sections, see nature.com/documents/nr-reporting-summary-flat.pdf

Life sciences study design

All studies must disclose on these points even when the disclosure is negative.

Sample size	Sample sizes were calculated on the assumption that 20-30% difference in the parameters measured would be considered biologically significant with an estimate of sigma of 10-20% of the expected mean. Alpha and Beta were set to the standard values of 0.05 and 0.2, respectively.
Data exclusions	No exclusions.
Replication	All experiments reported in the manuscript were replicated to confirm reproducibility, and biological replicates were performed as stated in the figure legends.
Randomization	For all animal studies, the animals were randomized after selecting a group of sex-appropriate mice.<br>For immunohistochemistry/immunofluorescence, the slides for each specific groups were randomized after sample processing.
Blinding	µCT analysis, cell assays, and immunohistochemistry/immunofluorescence were performed by investigators who were blinded under analysis.

Reporting for specific materials, systems and methods

We require information from authors about some types of materials, experimental systems and methods used in many studies. Here, indicate whether each material, system or method listed is relevant to your study. If you are not sure if a list item applies to your research, read the appropriate section before selecting a response.

Materials & experimental systems

n/a	Involved in the study
☐	☒ Antibodies
☐	☒ Eukaryotic cell lines
☒	☐ Palaeontology and archaeology
☐	☒ Animals and other organisms
☒	☐ Clinical data
☒	☐ Dual use research of concern
☒	☐ Plants

Methods

n/a	Involved in the study
☒	☐ ChIP-seq
☐	☒ Flow cytometry
☒	☐ MRI-based neuroimaging

Antibodies

Antibodies used	The detailed information of all antibodies (supplier name, catalog name, clone name and RRID) could be found in SI, KEY RESOURCES TABLE.
Validation	The following validation methods were conducted: 1) isotype controls were used, 2) for IHC/IF, the secondary fluorescent antibody was added alone without the primary antibody, 3) the manufacturer has validated these antibodies for use in the same species and assay format.

Eukaryotic cell lines

Policy information about cell lines and Sex and Gender in Research

Cell line source(s)	Validation of cellular senescence in different Craniosynostoses was initially performed using MC3T3-E1 cells from ATCC (CRL-2594), a commercially available mouse osteoblast precursor cells, which was established from the skull of C57BL/6 mice.
Authentication	ProCell performed authentication. The cell line was not authenticated by the authors.
Mycoplasma contamination	All cells were confirmed to be free of mycoplasma.
Commonly misidentified lines (See ICLAC register)	No commonly misidentified cell lines were used.

Animals and other research organisms

Policy information about studies involving animals; ARRIVE guidelines recommended for reporting animal research, and Sex and Gender in Research

Laboratory animals	In this study, Fgfr2C361Y/+ mice were engineered to carry the Cys361Tyr point mutation (correrspond to the human Cys342Tyr mutation) based on the Fgfr2-215 transcript (ENSMUST00000122054.8).11 The mouse model was generated by GemPharmatech (Nanjing, China) using CRISPR/Cas9 technology, with synonymous mutations introduced at site 362 to avoid secondary cleavage. For the construction scheme and validation, see Figure S1b and Table S1. Mapk14-KO mice (Mapk14-/+, Cat. no. NM-KO-225013) and Mapk14-Flox mice (Mapk14f/f, Cat. NO. NM-CKO-2102209) were purchased from Southern Model Organisms (Shanghai, China). Prrx1-Cre mice (Prrx1cre, Cat. NO. 005584) was purchased from The Jackson Laboratory. Fgfr2C361Y/+; Mapk14-/+ mice, Fgfr2C361Y/+; Prrx1cre/+; Mapk14f/+ mice and Fgfr2C361Y/+; Prrx1cre/+; Mapk14f/f mice were obtained via hybridization between the individual strains. All mice were maintained on a C57BL/6J background throughout the study period.
Wild animals	This study did not involve wild animals.
Reporting on sex	Sex was not considered in study design.
Field-collected samples	This study did not involve field-collected samples.
Ethics oversight	All animal experiments and procedures were conducted in accordance with the National Institutes of Health Guidelines for the Care and Use of Laboratory Animals.

Note that full information on the approval of the study protocol must also be provided in the manuscript.

Plants

Seed stocks	N/A
Novel plant genotypes	N/A
Authentication	N/A

Flow Cytometry

Plots

Confirm that:

☒ The axis labels state the marker and fluorochrome used (e.g. CD4-FITC).

☒ The axis scales are clearly visible. Include numbers along axes only for bottom left plot of group (a 'group' is an analysis of identical markers).

☒ All plots are contour plots with outliers or pseudocolor plots.

☒ A numerical value for number of cells or percentage (with statistics) is provided.

Methodology

Sample preparation	Single-cell suspensions were prepared and washed 1–2 times with pre-cooled staining buffer (PBS containing 2% FBS and 1 mM EDTA), followed by blocking with Fc blocking buffer (Elabscience; 1:50) at 4°C for 20 minutes. DAPI staining solution (Thermo Fisher Scientific, 0.1 µg/100µl) was added, and the cells were incubated in the dark for 5 minutes. After washing twice with flow buffer, cells were incubated with the secondary antibody at 4°C for 20 minutes. After washing, the cells were analyzed or sorted. The SPC population was defined as CD45-Ter119-Tie-26C3-Thy1-CD105-CD51+CD200+. The SPC ratio was determined as the proportion of CD51+CD200+ cells within the single-cell population after de-adhesion and removing DAPI+ cells.
Instrument	Flow cytometry was performed using a BD FACSCelesta for analysis or a BD FACSAria III for sorting.
Software	The FlowJo software (v10.8.1) was used for data analyses.
Cell population abundance	Sorting was performed using a 4-way collection and it was validated to result in >95% purity of the intended population in post-sort fractions.
Gating strategy	Total dissociated cells were fractionated by side scatter (SPC) and forward scatter (FSC) to discriminate single cells versus debris and doublets. Single cells were then fractionated by Dapi- and negative lineage (CD45-, Ter119-, Tie2-, Thy1- and 6C3-). The negative population was then further fractionated by CD51 and CD105. CD105-/CD51+ population was gated against CD200 for final isolation of SPC (CD51+CD200+CD105-CD45-Ter119-Tie2-Thy1-6C3-).

☒ Tick this box to confirm that a figure exemplifying the gating strategy is provided in the Supplementary Information.
